# Oligomerization of Uukuniemi virus nucleocapsid protein

**DOI:** 10.1186/1743-422X-7-187

**Published:** 2010-08-10

**Authors:** Anna Katz, Alexander N Freiberg, Vera Backström, Axel R Schulz, Angelo Mateos, Liisa Holm, Ralf F Pettersson, Antti Vaheri, Ramon Flick, Alexander Plyusnin

**Affiliations:** 1 Department of Virology, Infection Biology Research Program, Haartman Institute, P.O. Box 21, 00014, University of Helsinki, Helsinki, Finland; 2 Department of Pathology, University of Texas Medical Branch, 301 University Boulevard, Galveston, TX 77555-0609, USA; 3 Structural Genomics Group, Institute of Biotechnology P.O. Box 56, 00014 University of Helsinki, Helsinki, Finland; 4 Ludwig Institute for Cancer Research, Stockholm Branch, Karolinska Institute, P.O. Box 240, 17177 Stockholm, Sweden; 5 Software Point, Valkjärventie 1, 02130 Espoo, Finland; 6 BioProtection Systems Corporation, 2901 South Loop Drive, Suite 3360, Bldg. 3, Ames, IA 50010-8646, USA

## Abstract

**Background:**

Uukuniemi virus (UUKV) belongs to the *Phlebovirus *genus in the family *Bunyaviridae*. As a non-pathogenic virus for humans UUKV has served as a safe model bunyavirus in a number of studies addressing fundamental questions such as organization and regulation of viral genes, genome replication, structure and assembly. The present study is focused on the oligomerization of the UUKV nucleocapsid (N) protein, which plays an important role in several steps of virus replication. The aim was to locate the domains involved in the N protein oligomerization and study the process in detail.

**Results:**

A set of experiments concentrating on the N- and C-termini of the protein was performed, first by completely or partially deleting putative N-N-interaction domains and then by introducing point mutations of amino acid residues. Mutagenesis strategy was based on the computer modeling of secondary and tertiary structure of the N protein. The N protein mutants were studied in chemical cross-linking, immunofluorescence, mammalian two-hybrid, minigenome, and virus-like particle-forming assays. The data showed that the oligomerization ability of UUKV-N protein depends on the presence of intact α-helices on both termini of the N protein molecule and that a specific structure in the N-terminal region plays a crucial role in the N-N interaction(s). This structure is formed by two α-helices, rich in amino acid residues with aromatic (W7, F10, W19, F27, F31) or long aliphatic (I14, I24) side chains. Furthermore, some of the N-terminal mutations (e.g. I14A, I24A, F31A) affected the N protein functionality both in mammalian two-hybrid and minigenome assays.

**Conclusions:**

UUKV-N protein has ability to form oligomers in chemical cross-linking and mammalian two-hybrid assays. In mutational analysis, some of the introduced single-point mutations abolished the N protein functionality both in mammalian two-hybrid and minigenome assays, suggesting that especially the N-terminal region of the UUKV-N protein is essential for the N-N interaction.

## Background

Uukuniemi virus (UUKV) belongs to the *Phlebovirus *genus in the family *Bunyaviridae*. Some members of the family are important human pathogens, e.g. Crimean-Congo hemorrhagic fever virus, hantaviruses, and Rift Valley fever virus (RVFV) [1]. UUKV was first isolated from ticks in Uukuniemi, Finland, in 1959 [2], and as a non-pathogenic virus for humans [3], UUKV has served as a safe model bunyavirus in a number of studies addressing fundamental questions, e.g. organization and regulation of viral genes, structure and assembly [4-7]. Like other *Bunyaviridae*, UUKV is an enveloped virus with a tripartite RNA genome of negative polarity. The large (L) segment encodes the RNA-dependent RNA polymerase (L protein), and the medium (M) segment encodes two glycoproteins, G_N _and G_C_. The small (S) segment encodes the nucleocapsid (N) protein and, in positive sense orientation, the non-structural protein [1]. N protein plays a central role in the replication, transcription and assembly of RNA viruses. In negative-strand RNA viruses (NSRV), including bunyaviruses, both the vRNA and cRNA are encapsidated by the N protein into a ribonucleoprotein (RNP) complex, which serves as template for transcription and replication of the viral genome [8]. In the course of RNA encapsidation, the N protein of NSRV forms oligomers.

Among the NSRV, this oligomerization ability has been demonstrated for several viruses, for example Marburg virus (*Filoviridae*) [9], Sendai virus (*Paramyxoviridae*) [10], and influenza A virus (*Orthomyxoviridae*) [11]. In addition, N protein 3D-structures for four viruses were solved recently - rabies and vesicular stomatitis viruses (*Rhabdoviridae) *[12,13], Borna disease virus *(Bornaviridae) *[14], and influenza A virus [15] - revealing the oligomerization domains in detail.

The ability of N protein to oligomerize has also been shown for bunyaviruses in different genera: Bunyamwera virus (BUNV) (*Orthobunyavirus*) [16], hantaviruses (*Hantavirus*) [17,18], tomato spotted wilt virus (*Tospovirus*) [19], and RVFV (*Phlebovirus*) [20]. Throughout the five genera, the sizes of bunyaviral N proteins differ from 25 to 30 kDa (orthobunya-, phlebo-, and tospoviruses) to double the size, 48 to 54 kDa (hanta- and nairoviruses). The mode of N protein oligomerization seems to differ between the genera as well. BUNV-N protein was shown to form dimers, trimers and higher multimers [16,21], and Tula hantavirus N protein to form oligomers through trimer formation, where the N-terminal coiled-coils are involved [22-25]. These coiled-coiled domain structures have also been solved for two hantaviruses, Sin Nombre virus and Andes hantavirus [26,27]. A head-to-head and tail-to-tail fashion of oligomerization was suggested for both BUNV and Tula hantavirus N proteins. For RVFV-N protein, dimer formation was suggested, and the N-N interacting domain was mapped to the first 71 N-terminal residues [20]. Further details of the oligomerization process remain largely unknown.

In the present study, we focused on the oligomerization of the UUKV-N protein. Our first experiments using the mammalian two-hybrid (M2H) system and chemical cross-linking showed that the UUKV-N protein molecules can interact with each other. The aim was then to locate domains involved in the N protein oligomerization and to study this process in more detail.

## Results

### Analysis of UUKV-N protein in the chemical cross-linking and M2H assays

**Figure 1:**
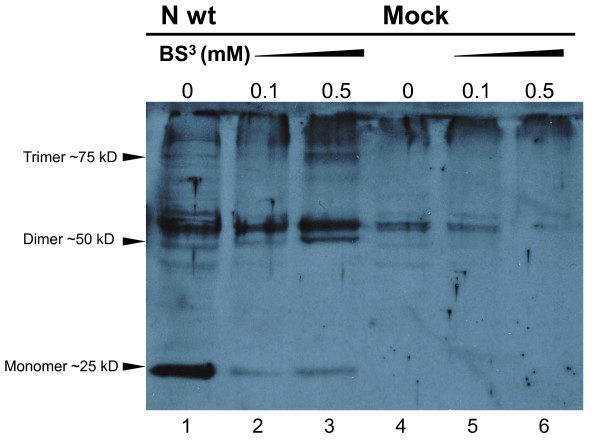
**Cross-linking of the UUKV-N protein**. N protein containing COS-7 cell lysates were treated with 0.1 or 0.5 mM of cross-linking reagent (BS^3^), and resolved by 10% SDS-PAGE under denaturing conditions. An immunoblot shows that omitting BS^3^, the majority of the full-length UUKV-N protein migrated as 25 kDa monomeric form (lane 1). Addition of BS^3 ^decreased the amount of the monomeric form, while the amount of dimeric and multimeric forms were simultaneously increasing (lanes 2 and 3). In mock-transfected samples no N protein was detected (lanes 4 to 6).

To study the N protein oligomerization, COS-7 cells were transfected with pcDNA-UUKV-N constructs and lysates were treated with the chemical cross-linker, BS^3 ^(Fig. 1). In the absence of BS^3^, the monomeric form of the N protein (~25 kDa) was predominant in immunoblotting (Fig. 1, lane 1). In the presence of BS^3^, the intensity of monomeric band decreased and the dimeric form (~50 kDa) was formed. In the lysates treated with 0.5 mM of BS^3 ^also a band of ~75 kDa appeared, suggesting the presence of trimers (Fig. 1, lane 3). MOCK-transfected COS-7 cells served as control (Fig. 1, lanes 4 to 6). A band just above the N-dimer as well as the bands that move slower than the N-trimer probably presented unrelated cross-reacting cellular proteins.

These results were in agreement with those obtained using the M2H assay: UUKV-N protein was expressed as a fusion with the DNA-BD and DNA-AD domains, resulting in high luciferase reporter signal. Same M2H vectors without N protein inserts served as negative controls, leading to very low signals (data not shown). Thus, both assays clearly demonstrated the N protein ability to form dimers and higher oligomers.

### Secondary and tertiary structure predictions for UUKV-N protein

**Figure 2:**
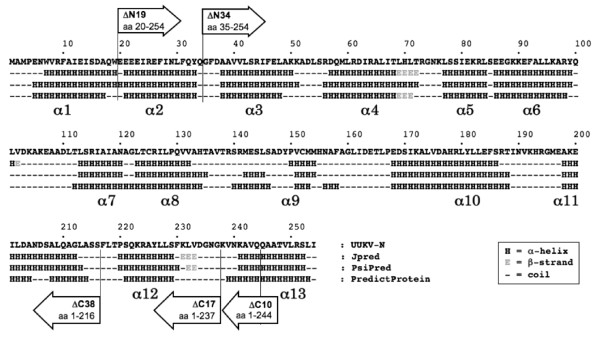
**Secondary structure predictions for UUKV-N protein (Jpred, PsiPred and PredictProtein servers) and mutagenesis strategy**. To study the oligomerization ability of UUKV-N protein, five deletion mutants were designed: ΔN19, ΔN34, ΔC38, ΔC17, and ΔC10.

For the secondary stucture, Jpred, Psipred and PredictProtein programs predicted the UUKV-N (254 aa residues) to be an α-helix-rich protein containing 13 to 15 α-helices with one to three short β-strands. Only minor differences in the length and location of the α-helices were observed between these models. In our working model we adopted 13 α-helices constantly predicted by all used programs (Fig. 2). For the N-terminal region the predictions showed either one long α-helix (aa residues 5-33), or two shorter α-helices (aa residues 7-19/5-17 and 21-33). In the C-terminal region, two α-helices (aa residues 220/221-229/230 and 239/241-252/253) were predicted (see α1, α2, α12 and α13 in Fig. 2).

**Figure 3:**
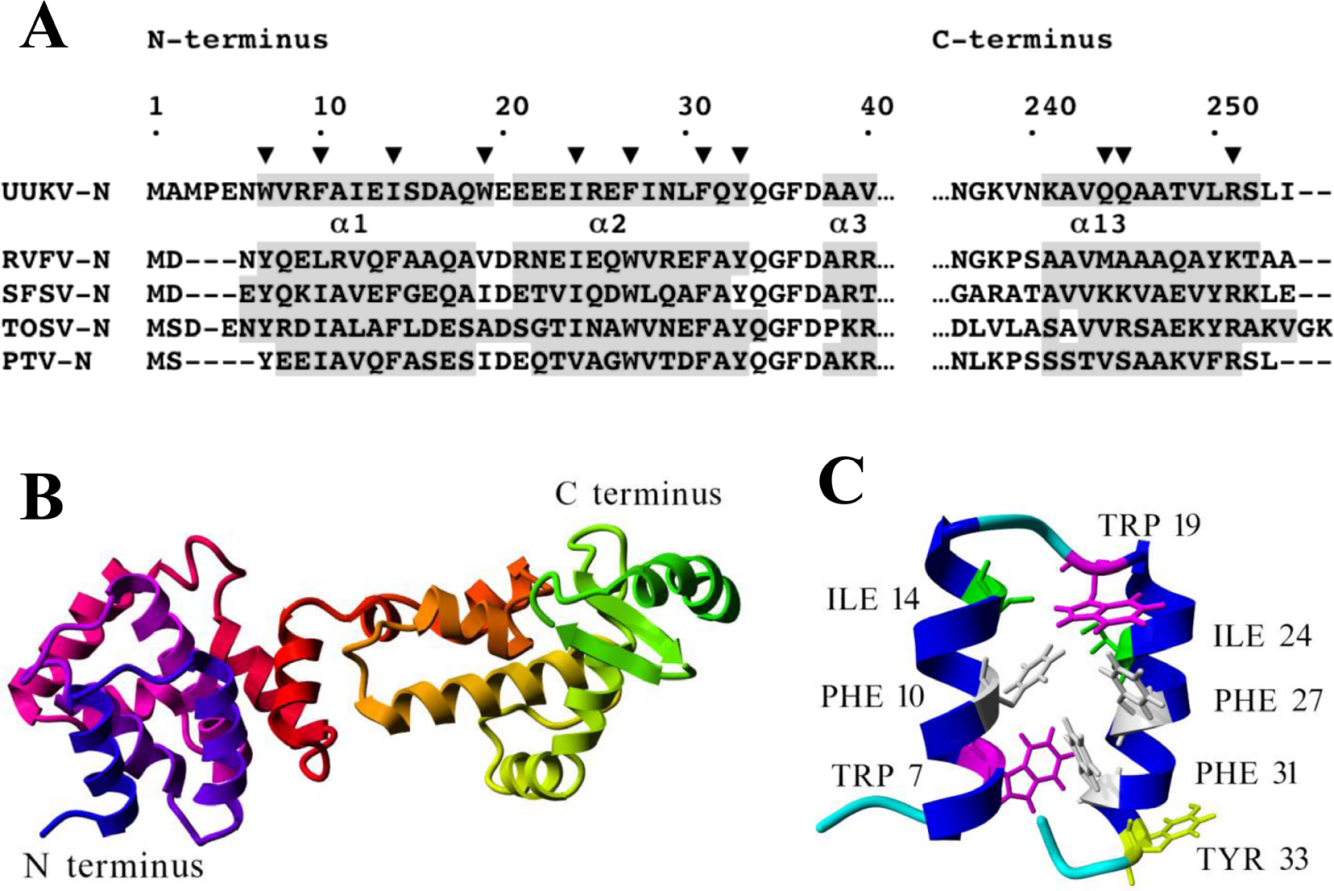
**Design for the UUKV-N protein mutations based on multiple alignments and 2D- and 3D-structure predictions.** (A) Jpred alignment shows the N- and C-termini of N proteins among five members in the genus Phlebovirus. α-helix-forming aa residues are shadowed. Point mutations for the UUKV-N protein were targeted to the aromatic and hydrophobic aa residues. (B) Robetta server's ab initio model for the UUKV-N  protein: the first two predicted α-helices in N-terminus are shown in blue, and the C-terminus is shown in green. (C) The N-terminal part of the Robetta model (Fig. 3B) showing the residues presumably involved in the oligomerization (except Y33 facing outside of the α-helices).

Next, the secondary structure predictions for UUKV-N were compared to N proteins of four other phleboviruses (RVFV, SFSV, TOSV and PTV). Predictions by the Jpred and PsiPred programs showed that the overall secondary structure is well conserved among phleboviruses (data not shown). For the N-terminus, predicted α-helices were of the same length (α1: 11-15 residues; α2: 12-13 residues) and located at similar positions (Fig. 3A). The same was true for the last C-terminal α-helices: α13 and α12 were predicted to be respectively, 11-14 and 10-11 aa residues long, with a short β-strand located between them. Shorter α-helices were predicted within the central part of the molecule, with less uniform pattern than in the well-conserved N- and C-terminal parts. This analysis suggested that conserved α-helices perform an important, fundamental function shared by all analyzed phleboviral N proteins.

Tertiary structure of the UUKV-N protein was predicted using Robetta server's *ab initio *modeling. Altogether, ten models were obtained, one of them is shown in Fig. 3B. All showed the same overall pattern of folding and, in agreement with the secondary structure predictions, contained 13-16 α-helices. Similarly to the PsiPred prediction, all Robetta models showed two α-helices separated by 2-3 aa residues at the N-terminal part of the molecule. At the C-terminal region, two antiparallel β-strands were predicted (Fig. 3B).

These models of UUKV-N protein were used to define our mutagenesis strategy. Since earlier studies on hantaviruses, RVFV, and BUNV showed that the very terminal regions of the N protein are especially important for the oligomerization [16,20,23,25], we focused on these regions.

### Analysis of the N-N interactions in M2H and minigenome assays

First, predicted α-helices were gradually removed from the N- and C-termini of the N protein molecule generating five deletion mutants: ΔN19, ΔN34, ΔC38, ΔC17, and ΔC10 (Fig. 2). In mutant ΔN19, the first α-helix, and in mutant ΔN34, the two first α-helices were deleted. Similar strategy was implemented for the C-terminus; in the mutant ΔC10, half of the last α-helix was removed, in mutant ΔC17 the entire last α-helix, and in mutant ΔC38 the last two α-helices were deleted (Fig. 2). These mutants were tested in the M2H system, and further studied using the minigenome system, immunofluorescence and cross-linking assays.

**Figure 4:**
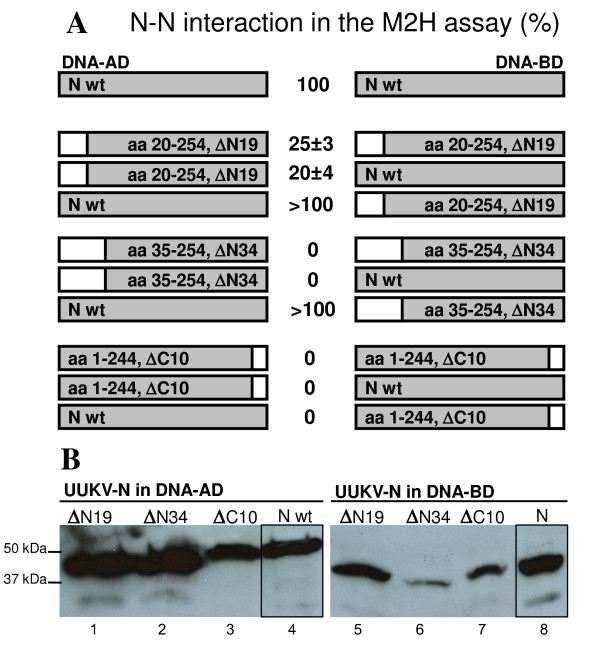
**N-N interaction of truncated UUKV-N protein constructs in the M2H assay and verification of the protein expression.** (A) Truncation ΔN19    affected the N-N interaction, ΔN34 and C-terminal truncation ΔC10    destroyed the oligomerization ability completely. Deleted regions are    shown in white. Numbers are averages of normalized luciferase activity    values (%), where the wt N-N interaction was set as 100%. ± standard    deviations are calculated for the mean values. (B) N protein    expression of the M2H constructs was verified in immunoblotting. MAbs    were used to detect the N protein fusions with DNA-AD (lanes 1 to 4),    and DNA-BD (lanes 5 to 8) constructs, which migrated as 44-46 kDa bands.

In the M2H system the full-length N protein showed strong N-N interaction ability (Fig. 4A). This ability decreased in the mutant ΔN19, where the remaining activity was only 25 ± 3%, and totally vanished in the mutant ΔN34 (Fig. 4A). This effect was repeatedly seen with DNA-AD-fused truncated proteins, even if they reacted with the full-length DNA-BD-N fusion protein. Two constructs, ΔN19-DNA-BD and ΔN34-DNA-BD, showed artificially high signals in the luciferase reporter assay, perhaps due to misfolding of the N fusion protein. All three C-terminal mutants, ΔC10 (Fig. 4A), ΔC17 and ΔC38 (data not shown) were completely non-functional, even if introduced in only one interacting partner (DNA-AD). To exclude the possibility that the lack of reactivity in the M2H assay was due to inefficient protein production, expression levels of truncated N proteins in COS-7 cells were confirmed by immunoblotting. Even though there was variation in the expression levels, it could not explain the differences in the M2H results (Fig. 4B).

**Figure 5:**
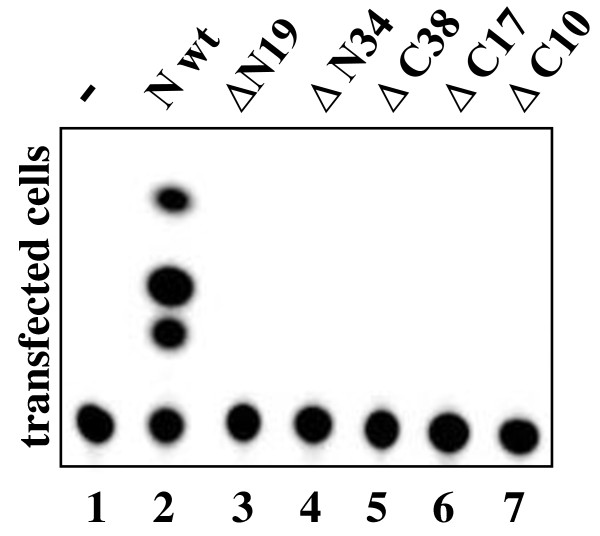
**Comparative CAT analysis on N protein deletion mutants**. BHK-21 cells were transfected with UUKV minigenome plasmids: (UUKV M-CAT), viral polymerase expression (pCMV-UUKV-L) and wt or mutant pcDNA-UUKV-N. Cells were analyzed for CAT activity at 48 h post-transfection. The N- and C-terminal deletion mutants (lanes 3 to 7) were compared with wt N protein (lane 2), showing that all the deletion mutants were non-functional in the minigenome system. In the negative control (lane 1) pCMV-UUKV-L was omitted.

**Figure 6:**
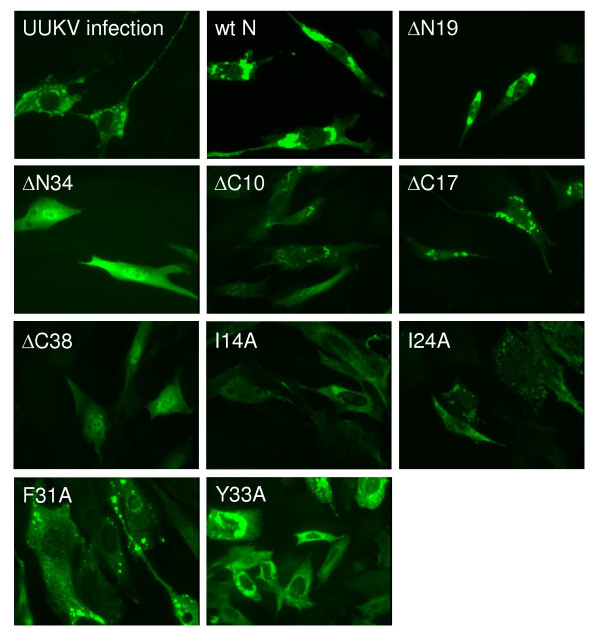
**Immunofluorescence analysis on the UUKV-N protein mutants at 24 h post-transfection**. The panel of UUKV-N deletion and point mutants shows that the intracellular localization and the oligomerization ability of some mutants (e.g. ΔN34 and ΔC17) was altered compared to the wt N protein and UUKV infected BHK-21 cells.

Deletion mutants were further tested in the UUKV minigenome system. The N protein molecules supposedly interact with each other and also with viral genome segments and replication intermediates and form RNPs. Interrupting the ability to form N-oligomers should interfere with minigenome transcription and replication. All five N- and C-terminal deletions completely abolished the function of the N protein, resulting in a negative CAT signal (Fig. 5). Mutants ΔN19 and ΔC10 were negative in CAT assays; this suggested that both terminal moieties are needed for the oligomerization process. Expression of the UUKV-N mutants was verified by immunofluorescence assay (IFA) (Fig. 6) and by immunoblotting (data not shown). Thus the results obtained with the minigenome system were in agreement with those of the M2H assay: both N- and C-terminal α-helices are essential for the N-N interactions and even small deletions completely destroyed the protein function (Fig. 4A and 5). Furthermore, the results of the cross-linking assay confirmed that all N- and C-terminal deletions severely affected the ability of N protein to oligomerize (data not shown).

### Analysis of the intracellular localization and distribution of wt and truncated UUKV-N protein using immunofluorescence assay

Next, we examined where the wt and truncated UUKV-N protein localizes in transfected cells and whether there are differences in the staining pattern (Fig. 6). Wild type (wt) N protein localized in the cytoplasm and formed larger aggregates (Fig. 6), resembling UUKV-infected cells (Fig. 6), as also presented earlier [28]. Of the N-terminal truncations, ΔN19 did not have a major effect on the appearance of the stained protein aggregates. It resembled the pattern of wt N protein, although some diffuse staining was observed. However, truncation ΔN34 differed from the wt N protein remarkably: the protein was dispersed throughout the cytoplasm as a diffuse net, with only a few microgranular aggregates observed (Fig. 6). In all C-terminal truncations, both intracellular localization and the staining pattern of the N protein were strongly affected. These truncated N proteins were dispersed as a diffuse pattern in the cytoplasm, with some small microgranular aggregates similar to mutant ΔN34. Truncated proteins were also observed in the nuclei, the effect was most pronounced with the longest truncation, ΔC38 (Fig. 6). Most importantly, no protein aggregates, characteristic of the wt N protein, were seen.

### Bioinformatic analysis and mutagenesis strategy of point mutations

**Figure 7:**
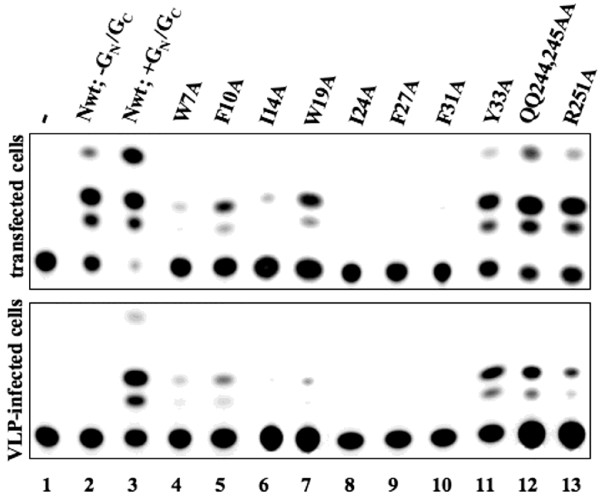
**UUKV-N point mutants analyzed by comparative CAT analysis and VLP transfer of UUKV M-CAT minigenome.** Upper panel: In comparative CAT analysis, BHK-21 cells were transfected with UUKV M-CAT, pCMV-UUKV-L and wt or mutant pcDNA-UUKV-N plasmids, and the glycoprotein expression plasmid pCMV-UUKV-G_N_/G_C_ was co-transfected for VLP transfer of the minigenome. Cells were analyzed for CAT activity, and VLP-containing supernatant was used to infect new cells pre-transfected with pCMV-UUKV-L and wt UUKV-N protein. Lower panel: UUKV-N point mutants analyzed by CAT expression and VLP transfer of UUKV M-CAT minigenome. Negative controls omit either pCMV-UUKV-L (lane 1) or include pCMV-UUKV-L but omit pCMV-UUKV-G_N_/G_C_ (lane 2; negative control for VLP transfer).

Experiments with truncated UUKV-N proteins directed us to focus on the N- and C-termini of the molecule, using site-directed mutagenesis to define aa residues involved in the N-N interactions. Sequence alignment and secondary structure predictions revealed very few conserved aa residues within the last C-terminal α-helix. To check whether this helix is directly involved in the N-N interaction(s) two mutations were introduced: R251, conserved in all phleboviruses (except K in RVFV), was replaced with alanine, and the double mutant QQ244,245AA, was designed to evaluate the possible role of the polar side chains in the N-N interaction. These mutants did not differ from the wt N protein in M2H, minigenome and VLP assays (Table 1, Fig. 7, lanes 12 and 13), and also in cross-linking and IFA (data not shown). It seems that the last C-terminal α-helix is not directly involved in the oligomerization but is indispensable for maintaining a proper folding of the whole molecule and hence its functional competence. We therefore concentrated on the N-terminal part of the protein.

**Table 1:** Summary of the results: UUKV-N protein point mutants in the M2 H, minigenome, and immunofluorescence assays.

UUKV-N protein point mutants	M2H* % of interaction		Minigenome^†^	VLP^†^	IFA^‡^
Wt N protein	100	+++	+++	+++	+++
W7A	> 100 (3)	+++	-	-	+++
F10A	34 ± 4 (2)	+	+	+	+++
I14A	60 ± 18 (3)	++	-	-	+
W19A	> 100 (3)	+++	+/++	-	+
I24A	56 ± 12 (2)	++	-	-	+
F27A	> 100 (3)	+++	-	-	++
F31A	23 ± 5 (2)	+	-	-	++
Y33A	> 100 (2)	+++	+++	+++	+++
QQ244,245AA	> 100 (2)	+++	+++	+++	+++
R251A	96 ± 11 (2)	+++	+++	+++	+++

* Full-length N-N protein interaction (100%, +++) was compared to the N-N interaction of point mutants, range from not affected (+++) to reduced (++) and to substantially reduced interaction (+). Each test was performed in triplicates, number of repetitions for each test is given in parentheses.

^† ^In minigenome and VLP systems the CAT expression was either non-affected (+++), reduced (++), substantially reduced (+), or completely inhibited (-).

^‡ ^Point mutants forming aggregates resembling full-length N protein (+++), showing microgranular (++) or diffuse (+) fluorescence pattern.

Most models indicated that the N-terminal region (aa 1-33), forms two α-helices separated by a short turn (Fig. 3C). This region is rich with aromatic and hydrophobic residues. In Robetta 3D-model of UUKV-N protein (Fig. 3B and 3C), it was observed that W7, F10, I14, W19, I24, F27 and F31 are facing the same side of a α-helical projection of the molecule. This structure with two parallel α-helices resembles the N-terminal coiled-coil structure of the hantaviral N protein that was shown to be important for the N-N interactions [23,25,29]. The model for UUKV-N protein suggests that the shared hydrophobic space between first two α-helices is not exposed to the solvent. After a conformational change opening the structure, the listed aa residues could interact with their partners in the other N-monomer. To evaluate the contribution of these residues to the N-N interaction, and the overall functionality of the N protein molecule, eight point mutants were generated: W7A, F10A, I14A, W19A, I24A, F27A, F31A, and Y33A (Fig. 3A, arrowheads). Tyrosine at position 33 was facing outside of the α-helices, and therefore its replacement with alanine was expected to have no effect on the oligomerization.

### Analysis of the N-terminal point mutations in M2H and minigenome assays

Eight alanine substitutions were introduced to the N protein fused with the DNA-BD and DNA-AD for M2H assay and pcDNA-UUKV-N expression plasmids. In M2H assay, four mutants, F10A, I14A, I24A, and F31A, showed a reduced N-N interaction ability. Four other mutations, W7A, W19A, F27A, and Y33A (our negative control), acted as the wt N protein (Table 1). In the minigenome system, five mutations (W7A, I14A, I24A, F27A, and F31A) completely abolished CAT expression, mutations F10A and W19A had a moderate impact, whereas mutation Y33A did not affect the functionality of the protein (Fig. 7, upper panel, and Table 1). Expression of all mutants was verified by IFA (Fig. 6) and immunoblotting (data not shown).

### Analysis of the point mutations in the virus-like particle (VLP) system

To further test point mutations on the N protein functional competence, we used recently developed infectious VLP system for UUKV [30], in which cells transfected with expression plasmids encoding for UUKV-G_N_/G_C_, N protein, and viral polymerase, together with the UUKV minigenome, generate VLPs containing the minigenome. UUK-VLPs are released into the cell supernatant and able to infect new cells. It was assumed that if the N protein functionality is affected, it would inhibit, or even abolish, both packaging and transfer ability of the minigenome. No CAT activity was detected in the negative control of VLP-infected cells omitting UUKV-L and UUKV-G_N_/G_C_, respectively (Fig. 7, lower panel, lanes 1 and 2). The positive control containing UUKV-G_N_/G_C _showed strong CAT activity (Fig. 7, lower panel, lane 3). Six N-terminal point mutants (W7A, I14A, W19A, I24A, F27A, and F31A) showed reduced CAT signal indicating the affected N protein ability to oligomerize and/or encapsidate minigenome RNA (Fig. 7 and Table 1).

Three of these mutants, I14A, I24A, and F31A, showed reduced functional competence in the minigenome assay and affected N-N interaction ability in the M2H assay. In addition, two mutants, W7A and F27A, showed either reduced or totally inhibited functional ability in the minigenome assay and in the M2H assay gave artificially high signals. This discrepancy could indicate on a possible involvement of these residues in RNA-binding. Our control (Y33A) as well as two C-terminal mutants (R251A and QQ244,245AA) acted similarly in the VLP-assay compared to the wt N protein, i.e. were able to encapsidate, package and transfer a functional minigenome. To summarize, the results of the VLP assay were in agreement with the other two tests described above. They also logically showed that the demand for the functional competence of the involved components, including the N protein, is higher in this more integral system thus fewer alterations are tolerated.

### Immunofluorescence microscopy of UUKV-N protein mutants

All eight N-terminal and two C-terminal point mutations were also tested in IFA. Six mutants, including our negative control and two C-terminal mutants (W7A, F10A, F27A, Y33A, QQ244,245AA, and R251A) behaved as the wt N protein. They formed aggregates located mostly in the perinuclear region (a typical mutant from this group, Y33A, is shown on Fig 6). In sharp contrast, four other mutants (I14A, W19A, I24A, and F31A) showed a diffuse to microgranular pattern of staining (Fig. 6 and data not shown). These results suggested that at least some of the mutations, which inflicted the N-N interactions, also affected the intracellular localization of the N protein.

Results of different assays are summarized in Table 1. For six mutants the correlation was good. These mutations included those that affected the N protein functionality (I14A, I24A and F31A), and also the ones inflicting no detectable damage (Y33A, QQ244,245AA, R251A). The results for other mutations were ambiguous. Mutants W7A and F27A were particularly interesting: although they were capable to interact in the M2H assay and their IFA-staining pattern was the same as of the wt N protein, these mutations were not functional in the minigenome assay.

## Discussion

Results presented in this paper show that the molecules of UUKV-N protein molecules can interact with each other. Thus, in this respect, the UUKV-N protein resembles the nucleocapsid proteins of other NSRV [9-14,16-19].

Our experiments revealed that the oligomerization ability of UUKV-N protein depends on the presence of intact α-helices on both termini of the molecule. Moreover, the point mutagenesis data in combination with the computer modeling, suggested that a specific structure in the N-terminal region plays a crucial role in the N-N interaction(s). This structure (Fig. 3C) is formed by two α-helices, rich in aa residues with aromatic (W7, F10, W19, F27, F31, Y33) or long aliphatic (I14, I24) side chains. Seven of these residues are predicted to face the same side of the α-helical structure and presumably form an interacting surface during the oligomerization process. The side chain of Y33 is oriented differently (Fig. 3C) and therefore this residue served as a useful control. Replacement of any of the seven above-mentioned aa residues with alanines significantly reduced the N protein functionality in at least one of three functional assays: M2H, minigenome or VLP assays and, as expected, the mutation Y33A did not affect the N protein functionality (Table 1). Note that the M2H assay is best suited for the direct evaluation of the N-N interacting ability, whereas the minigenome and VLP assays are more complex and thus more demanding in terms of functional competence of the N protein. It should be capable not only to oligomerize but also to interact with the RNA template, the cytoplasmic tail of G_N _protein and, perhaps with other components of a viral transcription-replication-packaging machinery such as the L protein. This could explain the results observed with mutants W7A and F27A: although they were capable to interact in the M2H assay and their IFA-staining pattern was the same as that of the wt N protein, these mutations were not functional in the minigenome assay suggesting that these aa residues might be involved in other functions, for example, RNA binding. Three of these seven mutants (I14A, I24A, and F31A) showed also a changed pattern of the intracellular distribution of N protein seen in the IFA. This diffuse or microgranular pattern in IFA staining most probably reflected a reduced oligomerization ability of the N protein molecule (Fig. 6).

Our data correlated well with the earlier observations made for another phlebovirus, RVFV. Le May *et al. *[20] mapped the N-N interacting domain to the first 71 N-terminal residues of RVFV-N protein and showed the particular importance of Y4 and F11 (corresponding to W7 and I14 of UUKV-N) for the oligomerization. Secondary structure predictions suggest a universal mode of folding for phleboviral N proteins, thus the oligomerization mechanism might be similar in all members of the *Phlebovirus *genus, and could also share some important features with other bunyaviral N proteins. Indeed, the very recent publication on structure of RVFV-N protein [31], suggest that due to high sequence identity, all phlebovirus N proteins have the same fold, which may also exist throughout the *Bunyaviridae *family.

Similarly to phleboviral N proteins, the N proteins of BUNV (genus *Orthobunyavirus*) and hantaviruses (genus *Hantavirus*) oligomerize in a head-to-head and tail-to-tail fashion, and in all three genera the N-terminus of the N protein plays an important role [16,21,22,25]. One would expect to see some differences between the genera as well. Indeed, in hantaviruses the C-terminal part of the N protein plays crucial role in the oligomerization thus even point mutations introduced to this region can totally destroy the functionality of the molecule [25]. In mapping of the BUNV-N protein [21] the N-N interacting residues were located to the N-terminal region, the middle region and the C-terminus of the N protein. In contrast, in RVFV-N protein the C-terminal region was not found essential for the N-N interaction [20] and our data on UUKV showed that point mutations supposedly destroying proper folding of the last C-terminal α-helix did not affect the N protein functionality (Fig. 7 and Table 1). Although deletion of the C-terminal α-helices resulted in loss of activity in the minigenome and M2H assays, this could be caused by overall misfolding of the truncated protein.

In this paper, 3D-model of UUKV-N protein predicted using an *ab initio *approach has been proven useful to direct experiments to analyze N-N interactions. In the absence of X-ray crystallography or NMR structures of UUKV-N protein, our 3D-model might be helpful also in studying other functions of the molecule, such as RNA binding that is highly relevant to the N protein oligomerization, since both processes are coupled. Studies on RNA-binding properties of UUKV-N protein go beyond the frame of this project. Our preliminary data on mapping the N protein RNA-binding domain confirmed that predictions drawn from the model are reasonably accurate [Katz et al., MS in preparation].

Details of the UUKV-N protein oligomerization remain largely unknown. As a working hypothesis one could consider two alternative modes of interaction between specific structures formed by the N-terminal α-helices: (1) The interaction occurs between intact structures; in this case two interacting surfaces that are formed by aromatic and long aliphatic side chains are coming into close proximity and form a shared hydrophobic space, and (2). The interaction occurs after a conformational change that opens the structure and forms a new interacting surface mainly, or even exclusively, from the same side chains. In the above mentioned work, Raymond and co-authors [31] showed the importance of the hydrophobic residues - both in maintaining the structural stability and as sites for the N-N interaction, as we suggest in our study.

## Conclusions

Our results show that UUKV-N protein has ability to form oligomers in chemical cross-linking and mammalian two-hybrid assays. This oligomerization ability depends on the presence of intact α-helices on both termini of the molecule. Moreover, a set of N protein mutations were analyzed in minigenome and mammalian two-hybrid assays; this data in combination with the computer modeling suggested that a specific structure in the N-terminal region plays a crucial role in the N-N interactions.

## Methods

### Viruses and cells

The origin and the preparation of the UUKV prototype strain S23 have been described earlier [32]. All cell lines were from the ATCC: BHK-21 cells were grown in Glasgow minimal essential medium (GMEM; Invitrogen), COS-7 cells in Dulbecco's modified Eagle medium (DMEM), HeLa cells in minimum essential medium (MEM), and Sf9 insect cells in SF-900 II SF medium (Invitrogen). The media were supplemented with 10% fetal bovine serum, 2 mM L-glutamine, 100 IU of penicillin/ml, and 100 μg streptomycin/ml and maintained at 37°C in a 5%-CO_2 _atmosphere.

### Antibodies and antisera

UUKV-N protein was detected with earlier described [28], or commercial (ProSci Inc.) rabbit polyclonal antibodies, and mouse monoclonal antibodies (MAbs) (R. F. Pettersson, unpublished data) against UUKV-N protein. The UUKV-N fusion proteins used in the M2H assay were detected using MAbs raised against GAL4 DNA-binding (DNA-BD) and/or VP16 DNA activation (DNA-AD) domains (Santa Cruz Biotechnology).

### Plasmids

UUKV-N mutants were derived from plasmid pGEM-3N [33] containing complete UUKV-N protein cDNA. The UUKV-N ORF was amplified using *Pfu *DNA polymerase (Fermentas), digested with the restriction endonucleases HindIII and XbaI, and cloned into pcDNA3.1(+) (Invitrogen), resulting in the construct pcDNA-UUKV-N encoding the full-length, wild type (wt) N protein. N- and C-terminal truncations were introduced by oligonucleotide-directed mutagenesis using primers carrying HindIII/XbaI restriction sites. These PCR products were also cloned into the plasmids used in the M2H assay: pM1, containing DNA-BD and/or pVP16 containing DNA-AD domain (BD Biosciences Clontech). Alanine substitutions were introduced into the plasmids with site-directed mutagenesis kit (Stratagene) according to the manufacturer's instructions. The accuracy cloning was verified by restriction analysis and sequencing.

### Structural analysis of UUKV-N protein and sequence alignments

The secondary structure of UUKV-N protein was predicted using servers Jpred http://www.compbio.dundee.ac.uk/~www-jpred/[34], PsiPred http://bioinf.cs.ucl.ac.uk/psipred/[35], and PredictProtein http://www.predictprotein.org/[36]. Tertiary structure of the N protein was predicted using Robetta server's *ab initio *modeling http://robetta.bakerlab.org/[37,38], and phlebovirus N protein sequences were aligned using ClustalW program http://www.ebi.ac.uk/clustalw.

### Chemical cross-linking

COS-7 cells were grown to 70-80% confluence and transfected with pcDNA-UUKV-N constructs and FuGene6 reagent (Roche Applied Science) according to the manufacturer's instructions. After 24 h cells were washed and scraped to PBS, pH 7.4, and freeze-thawed three times. Cell lysates were cross-linked using 0.1 and 0.5 mM bis[sulfosuccinimidyl] suberate (BS^3^) (Thermo Fisher Scientific) for 30 min at room temperature (RT). After quenching the reaction with 50 mM Tris-HCl, pH 7.5, proteins were separated on 10% SDS-PAGE. Proteins were detected by immunoblotting with a rabbit antiserum against UUKV-N protein [28], followed by incubation with horseradish peroxidase (HRP)-conjugated swine anti-rabbit IgG antibody (Dako) and visualized using the enhanced chemiluminescence (ECL) method.

### Mammalian two-hybrid (M2H) assay

HeLa cells were grown to 70-80% confluence and transfected with 0.5 μg of pM-UUKV-N and pVP-UUKV-N constructs expressing the full-length or mutated N protein, 0.5 μg of the reporter-encoded plasmid pG5luc expressing the firefly luciferase (FL, Promega), and 0.01 μg of pRL-SV40 expressing Renilla luciferase (RL, Promega) to normalize the results. Each mutant was tested in triplicate (2 μl FuGene6 reagent for each reaction), and all experiments were performed at least twice. The reporter gene activities were determined 24 h post-transfection with the Dual-Luciferase Reporter Assay System (Promega). To balance inherent variations in the M2H assay, the FL-values were normalized using the RL-values: Normalized value of experiment A = [(RL-value from N-N interaction/RL-value of experiment A) × (FL-value of experiment A)]. The formula for percent interaction = (normalized value of experiment A/normalized value of N-N interaction) × 100.

For immunoblotting, COS-7 cells were transfected with M2H constructs using the FuGene6 reagent, washed at 24 h, and lysed with the Passive Lysis Buffer (Dual-Luciferase Reporter Assay System) for 15 min on ice. Cell lysates were clarified and proteins were separated on 10% SDS-PAGE under reducing conditions, and transfered to nitrocellulose membrane. Proteins were detected by incubation with MAbs recognizing DNA-BD and DNA-AD domains, followed by incubation with HRP-conjugated rabbit anti-mouse IgG antibodies (Dako), and visualized using the ECL system.

### Immunofluorescence assay (IFA)

BHK-21 cells were grown on coverslips to 70-80% confluence, and either transfected with 0.5 μg of wt or mutant pcDNA-UUKV-N constructs with 2 μl of FuGene6 reagent, or infected with UUKV with undefined multiplicity of infection (estimate from 1 to 5) for 1 h, after which the medium was replaced. At 24 h post-transfection and infection, cells were washed and fixed with 3% paraformaldehyde (15 min) and permeabilized with 0.1% Triton X-100 (30 min), both in PBS. After washing, the coverslips were incubated with two UUKV-N MAbs (30 min), followed by FITC-conjugated rabbit anti-mouse IgG antibodies (Dako) (30 min), all incubations at RT. Images were collected with Zeiss Axioplan 2 microscope.

### RNA Pol I-driven UUKV minigenome system

BHK-21 cells were grown to 80% confluence and transfected with UUKV M-CAT, pCMV-UUKV-L [5], and wt or mutated pcDNA-UUKV-N, using 4 μl of LipofectAMINE™2000 transfection reagent (Invitrogen) following the manufacturer's instructions. Cells were analyzed for reporter gene chloramphenicol acetyltransferase (CAT) activity 48 h post-transfection. The CAT assay was performed as described earlier [5], and according to the manufacturer's instructions (FAST CAT Kit; Invitrogen). Briefly, cells were harvested in PBS, resuspended in 250 mM Tris-HCl (pH 7.4) and lysed by three freeze-thaw cycles. Clarified cell lysates were mixed with 9 mM acetyl coenzyme A (Sigma Aldrich), Component A (Fast CAT Kit) and 250 mM Tris-HCl (pH 7.4). After 2 to 4 h at 37°C, samples were prepared for thin-layer chromatography and the reaction products were visualized by UV illumination.

### Virus-like particle (VLP) system for UUKV

VLP infection was performed as described earlier [30]: Briefly, supernatant of cells transfected with the above-mentioned minigenome plasmids and pCMV-UUKV-G_N_/G_C _expressing the UUKV M segment was transfered to BHK-21 cells. These cells were transfected with pCMV-UUKV-L and wt pcDNA-UUKV-N 24 h prior VLP passage to maintain replication and transcription of the transfered minigenome, thereby allowing expression of the reporter gene. After 1 h incubation the inoculum was replaced by fresh medium and cells were analyzed for CAT activity 48 h post-infection. The CAT assay was performed as described above. For immunoblotting, cells were lysed in the M-PER^® ^Reagent (Pierce), and incubated 20 min on ice. Cell lysates were separated by 10% SDS-PAGE and UUKV-N proteins were detected by immunoblotting with a rabbit polyclonal anti-UUKV-N antibody (ProSci), followed by incubation with HRP-labeled goat anti-rabbit IgG antibody (Sigma).

## Competing interests

The authors declare that they have no competing interests.

## Authors' contributions

AK and AP designed the study, AV, LH, RP and RF were involved in the study design. AK prepared recombinant plasmids and introduced all truncations and point mutations. AK, VB and LH performed bioinformatic analysis. AK performed chemical cross-linking, M2H assay, IFA, and immunoblotting for the M2H constructs; AF, ARS and AM performed the minigenome and VLP assays including the immunoblotting. AK, AF, VB, RF and AP analyzed the data. AK, AF and AP wrote the draft of the manuscript. All authors read and approved the final manuscript.
